# Intuitive human interface to a scanning tunnelling microscope: observation of parity oscillations for a single atomic chain

**DOI:** 10.3762/bjnano.10.33

**Published:** 2019-02-04

**Authors:** Sumit Tewari, Jacob Bakermans, Christian Wagner, Federica Galli, Jan M van Ruitenbeek

**Affiliations:** 1 Huygens-Kamerlingh Onnes Laboratory, Leiden University, Niels Bohrweg 2, 2333 CA Leiden, Netherlands; 2 current address: Department of Materials, University of Oxford, OX1 3PH, Oxford, United Kingdom; 3 current address: Nuffield Department of Clinical Neuroscience, University of Oxford, OX3 9DU, Oxford, United Kingdom; 4 Peter Grünberg Institut (PGI-3) Forschungszentrum Jülich, 52425 Jülich, Germany

**Keywords:** adatom imaging, mechanical annealing, scanning tunnelling microscopy (STM), STM tip, tip apex

## Abstract

A new way to control individual molecules and monoatomic chains is devised by preparing a human–machine augmented system in which the operator and the machine are connected by a real-time simulation. Here, a 3D motion control system is integrated with an ultra-high vacuum (UHV) low-temperature scanning tunnelling microscope (STM). Moreover, we coupled a real-time molecular dynamics (MD) simulation to the motion control system that provides a continuous visual feedback to the operator during atomic manipulation. This allows the operator to become a part of the experiment and to make any adaptable tip trajectory that could be useful for atomic manipulation in three dimensions. The strength of this system is demonstrated by preparing and lifting a monoatomic chain of gold atoms from a Au(111) surface in a well-controlled manner. We have demonstrated the existence of Fabry–Pérot-type electronic oscillations in such a monoatomic chain of gold atoms and determined its phase, which was difficult to ascertain previously. We also show here a new geometric procedure to infer the adatom positions and therefore information about the substrate atoms, which are not easily visible on clean metallic surfaces such as gold. This method enables a new controlled atom manipulation technique, which we will refer to as point contact pushing (PCP) technique.

## Introduction

It is of fundamental interest both for chemists and physicists to study the electronic transport through single atoms and molecules. Scanning tunnelling microscopy bestows us with the capability not only to image single atoms and molecules when they are deposited on a conducting surface but also to study electronic transport through these entities [1–2]. However, during atomic and molecular manipulation operations it is not possible to image the structural changes that happen at the junction using the STM, because the very STM tip used for imaging is also used for manipulation. It is known that the electronic transport of these nanoscale devices depends strongly on their structural conformations and coupling to the leads [3–4]. Therefore, the knowledge about the structure of the junction at the atomic scale is critical to the understanding of these transport measurements.

The system that we have developed addresses this problem. We have added a 3D motion control system to our STM that helps in making any required tip trajectory and combined it with a molecular dynamics (MD) simulator that simulates in real-time the manipulation process going on in the STM. The MD simulation not only provides information about the atomic scale structure of the junction, but also serves as a visual feedback to the operator in real-time who can then choose to make a desired trajectory for better control of the manipulation process. This is especially important in the case of 3D manipulation of single molecules and atomic chains, as there are no predefined accurate trajectories [5–6] that one can set to do those manipulations. Therefore an adaptable trajectory is the only solution where the operator can continuously communicate with the experiment through the real-time MD simulation and define the trajectory at will using the motion control system. This human–machine augmented system thus provides a far better control of the manipulation process and can moreover be used for 3D manipulation. Previously, for better control of atomic manipulations, an audible feedback has been used [7]. In this, the tunnel-current signal is amplified and put on headphones, so that one hears a “doink” when the atom hops from one position to the next. This is certainly helpful, but it does not reveal where it has hopped, only that it hops. In this article first we will start with describing the experimental setup and sample preparation technique. Later in section “Real-time molecular dynamic simulation” we will discuss the main outline and assumptions made in preparing the real-time MD simulation. After that we report on using this system for a new lateral manipulation methodology that we refer to as point contact pushing (PCP) technique, followed by a 3D trajectory that enabled us to lift in a controlled way a chain of gold atoms above a metal surface. These atomic chains are known to show parity oscillations in conductance [8] while going from even to odd number of atoms in the chain. We detect this phenomenon while controllably lifting the chain of atoms and putting it back on the surface.

## Experimental

**Figure 1:**
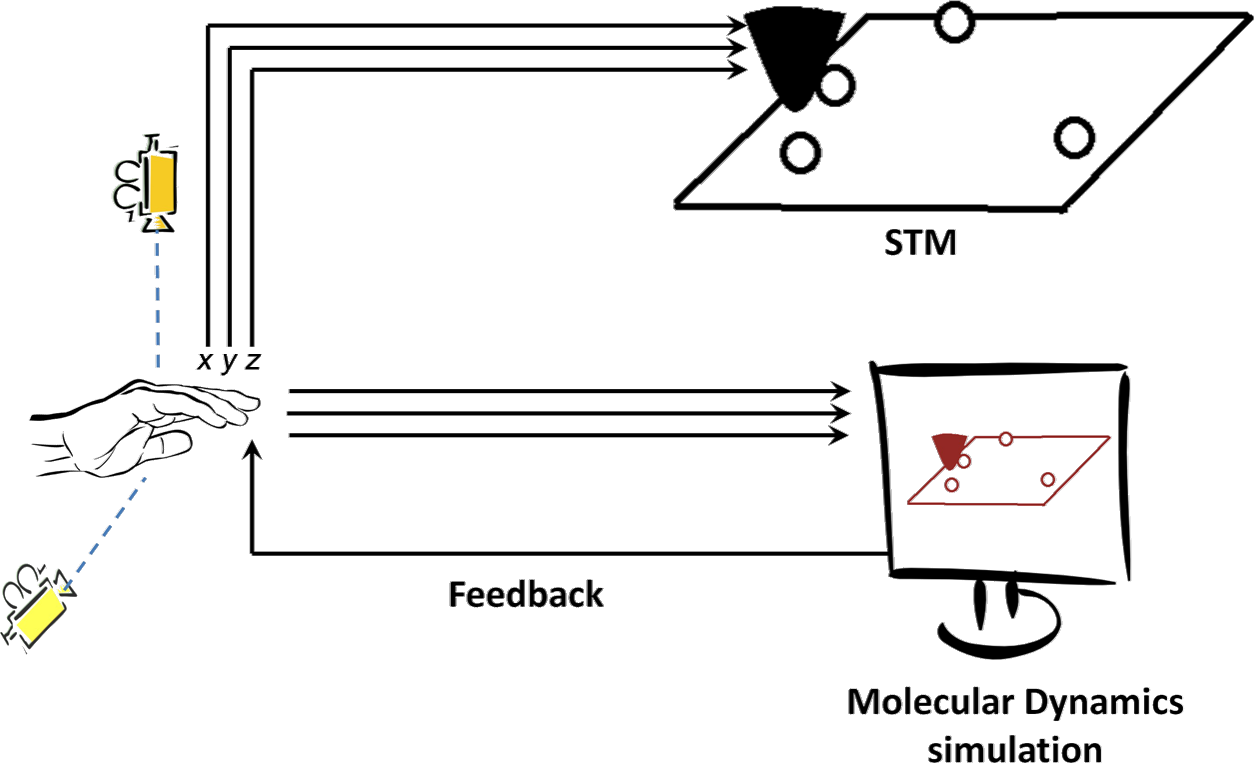
Schematic of the experimental setup. The components of the setup include a cryogenic UHV STM, a 3D motion controller and a MD simulator. The motion tracker controls both the STM and the MD simulation.

**Figure 2:**
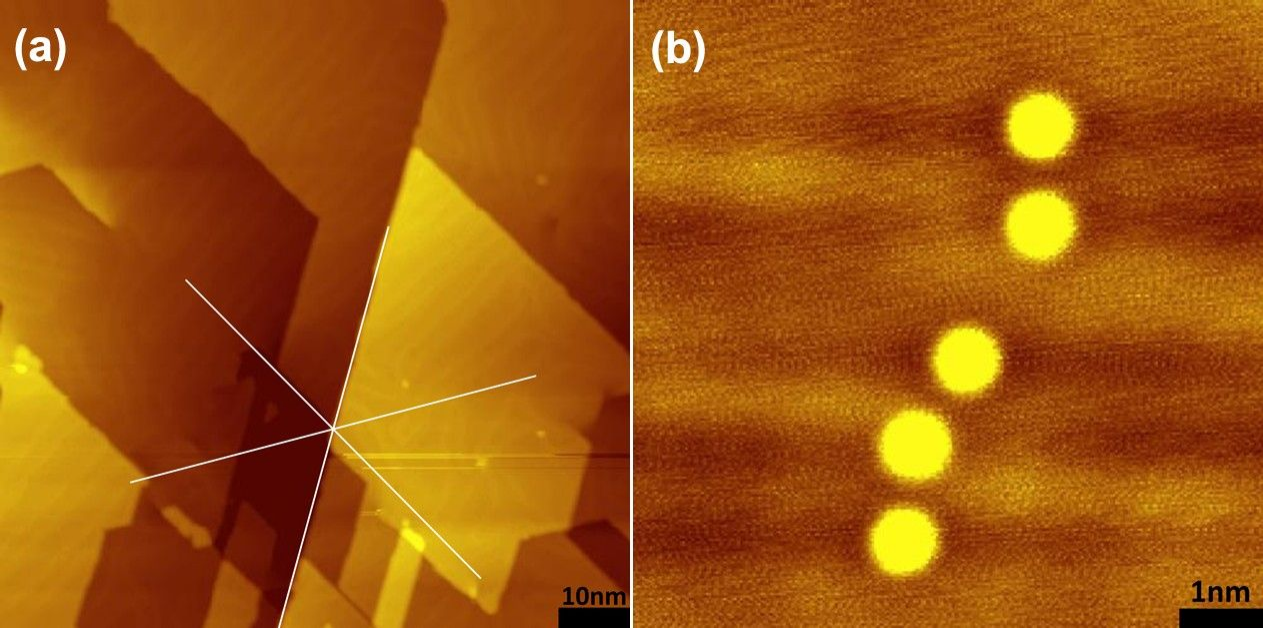
(a) Atomically flat Au(111) surface with herringbone reconstruction and straight step edges in crystallographic directions (i.e., 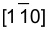, 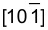 and 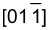) prepared by stress-induced lattice deformations at low temperatures, (b) Gold adatoms deposited on a Au(111) surface from the STM tip. Images obtained at about 3 K after the temperature was stabilized within a few millikelvins.

The experimental setup used here is a custom-built cryogenic STM head [9] that is cooled by a Oxford Heliox UHV system custom-built for Leiden [10]. The system operates at 10^−10^ mbar pressure and most of the experiments were performed at 3 K temperature (the base temperature of 300 mK was not required). A custom-built 3D motion-control system running under LabVIEW is used to control the STM tip in all three dimensions during manipulation. Figure 1 shows the schematic diagram of the complete setup with 3D motion controller and the MD real-time simulator. The 3D motion control system is an LED tracker made with two cameras tracking the *x*–*y*-motion and *y*–*z*-motion of the LED respectively. The LED is attached on top of the operator hand, such that the trajectory can be “drawn” by the operator hand and tracked by two cameras [5]. Then, a LabVIEW program filters and converts these *x*,*y*,*z*-signals with proper scaling factors before sending it to the STM tip and the simulation. The scaling factor converts approximately 10 cm of hand movement to 2 Å displacement of the STM tip. The usual imaging in STM is done using a commercial RHK SPM100 ver.8E controller. A mono-crystalline gold sample cut along the (111) surface is prepared by repeated argon sputtering and annealing cycles to obtain an atomically flat Au(111) facet showing herringbone surface reconstruction. We further prepare the surface at low temperature by creating a localized stress pattern [11–14] on the surface using gentle indentation of the STM tip at a spot on the surface remote from the area of investigation. This creates new crystalline (111) facets and provides straight step edges in the three crystallographic directions of Au(111) (i.e., 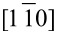, 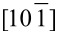 and 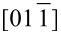) as shown in Figure 2a. Additional gold atoms (adatoms) are deposited [15–18] on the Au(111) surface at the target sites of investigation (Figure 2b) by establishing point contact with the surface using the STM tip at 100 mV bias. The STM tips used in the experiments are hand-cut PtIr tips that get covered by Au atoms on indentation of the surface.

## Real-time molecular dynamic simulation

**Figure 3:**
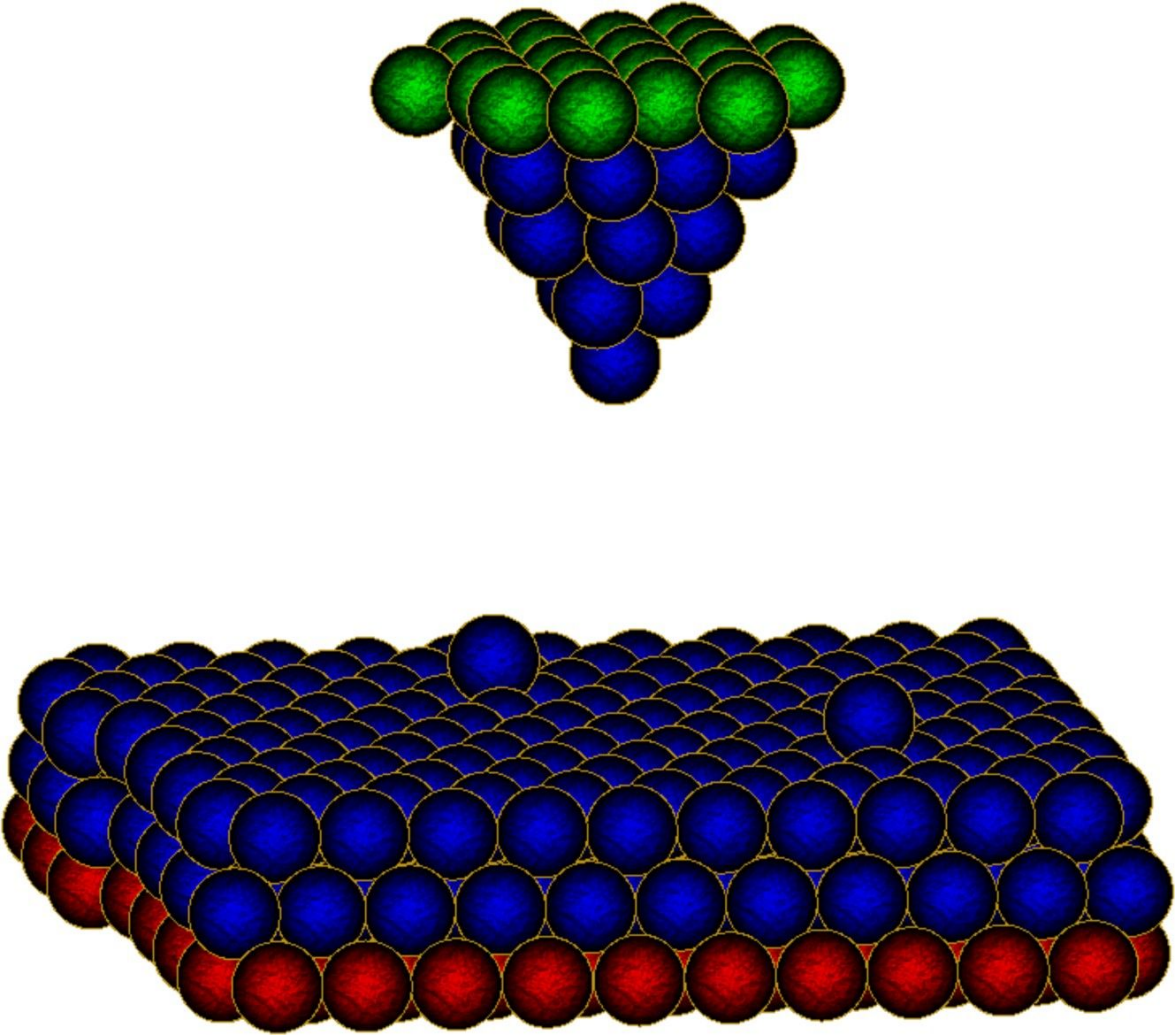
Snapshot of the molecular dynamics simulation showing the different atom types. ‘Normal’ gold atoms are drawn in blue, ‘tip boundary’ atoms in green and ‘surface boundary’ atoms in red.

A conventional atomic manipulation operation using STM involves a pre-defined trajectory (controlled by the operator or by an automated procedure) of the STM tip. An example is reducing the tip–sample distance and moving the tip in a desired direction assuming an isotropic nature of adsorption bonds [19] in metallic systems. In such a procedure the operator does not use any feedback from the current while the manipulation is executed and thus cannot influence the trajectory in order to respond to the complex dynamics of the tip/adatom/surface system during the manipulation. In contrast, in our setup the operator receives a continuous visual feedback from the real-time MD simulation. The visual feedback is in the form of a 2D projection of the 3D simulation output (as shown below in Figure 3) where the operator can see the position of all the atoms and their dynamics as the experiment proceeds. The operator can then respond to the predicted structural evolution of the junction during the manipulation operation and alter the trajectory at will. Figure 1 shows the scheme of communication between operator and STM using the MD simulator. The 3D motion tracking sensor sends the same *x*,*y*,*z*- signals to both the STM and the simulator simultaneously and therefore the MD simulation is required to have a minimal time delay in its response for smooth real-time operation. By ‘real time’ we mean that the system in the simulation converges to a local energy minimum (an equilibrium position) between each subsequent probe position. The probe speed is determined by the operator, and depends on the speed with which the 3D motion control sensor is moved. In the scale of the operator this is approximately 5 cm/sec, which corresponds to 1 Å/sec on the atomic scale.

We perform a classical MD simulation here in which we ignore the electronic effects (which in fact give rise to interatomic forces) and take the forces as coming from parameterised equations that only depend on the interatomic distances. This is typically called a force-field simulation. A more accurate method would be obtained by using ab initio calculations that take into account both the nuclear and the electronic degrees of freedom. But these ab initio calculations are computationally very expensive and thus are not suitable for our purpose. The simulation we discuss in this article is only made for metallic systems, so in this case all the atoms involved are Au atoms. Here a semi-empirical potential described by Tománek et al. [20] and Cortes-Huerto et al. [21] is used to model the Au–Au interaction. This allows for fast computation of a large number of atoms involved because of its simple analytical potential functions. The potential energy is given by

[1]
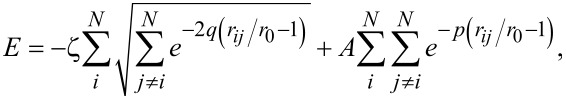


where *r_ij_* is the distance between two atoms *i* and *j*, *r*_0_ is the equilibrium distance, and ζ, *q*, *A* and *p* are parameters that can be determined by fitting bulk material properties to experimental values. The parameters used for the results shown in this manuscript are: *r*_0_ = 2.884 Å, ζ = 1.8184 eV, *A* = 0.20967 eV, *q* = 4.03 eV and *p* = 10.145 eV. The energy of Equation 1 consists of an attractive term (i.e., the energy decreases when the distance between two atoms decreases; this is the first term of Equation 1) and a repulsive term (the energy increases when the distance between two atoms decreases; this is the second term of Equation 1) [20]. The increase in kinetic energy for the conduction electrons confined between two approaching atoms gives rise to the repulsive term [22], while the attractive interaction originates from the band structure and is found by a second-moment approximation to the tight-binding Hamiltonian [20]. From this potential energy, forces can be calculated using

[2]
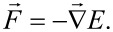


The force on an atom *a* is therefore given by (the derivation of this is given in Supporting Information File 1).

[3]
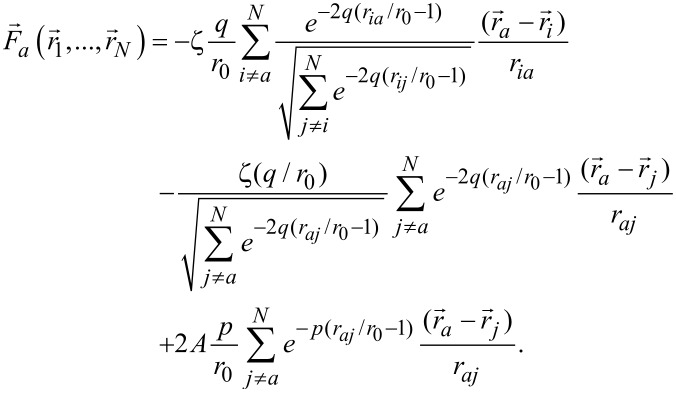


### Implementation

The molecular dynamics simulation is written in C++ to guarantee high computational performance. A schematic flowchart of the simulation execution is given in Supporting Information File 1 (Figure S4). Since providing visual feedback is one of the main objectives of the simulation, a graphics library is necessary to show visual output on the screen. For performance reasons and the ease of implementation the “Simple and Fast Multimedia Library” (SFML) [23] is used. We choose an object-oriented approach to keep the code well structured. One separate class is used to keep track of the individual atoms (i.e., storing and updating all the *r_ij_* values), another one to calculate energy and forces, and to integrate the equations of motion, and a third separate class to visualize the atoms.

We differentiate between three types of gold atoms, corresponding to the role they play in the simulation. In Figure 3, a snapshot of the simulation shows the different atom types. First, there are ‘normal’ gold atoms (drawn in blue) that only feel the forces of the other atoms through Equation 3. Then, there are ‘boundary’ atoms (drawn in green and red). These are gold atoms that are not entirely frozen [24] but feel an additional force to confine their positions. A 3D parabolic potential well for each boundary atom, centered at positions resembling a bulk lattice layer, keeps the metal slab and the tip in shape by fixing the boundaries. The potential wells mimic the presence of atoms beyond the boundaries. This approach allows for dynamics even for the boundary atoms, making it possible to apply a thermostat and have realistic interaction with the other normal gold atoms. There are two types of such boundary atoms: tip boundary atoms and surface boundary atoms. For surface boundary atoms, the position of the potential wells stays the same throughout the simulation. For tip boundary atoms, the position of the potential wells can be changed to simulate tip motion [24]. As there is a huge discrepancy in timescales between experiment and simulation, a tip motion of some angstroms in several seconds in experiments happens within picoseconds in the simulation, yielding a much higher tip velocity and acceleration in the simulation. This large amount of kinetic energy pumped into the system has to be drained out using a suitable thermostat. A Berendsen thermostat [25] is implemented into the simulation that provides a gradual temperature decay instead of sudden rescaling. Here the instantaneous temperature changes proportional to the temperature difference with the reference temperature *T*_0_ with an adjustable coupling to a heat bath:

[4]
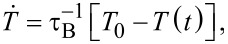


where τ_B_ is the temperature relaxation time, related to the strength of the coupling. The velocities of all atoms are rescaled at every timestep (Δ*t*) with the same factor:

[5]
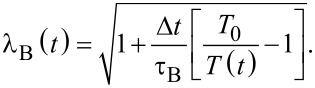


A typical value for τ_B_ in condensed systems is of the order of 0.1 ps [26]. In our case only the boundary atoms are subject to temperature control by a thermostat. This way kinetic energy is transferred through the normal atoms to the boundary atoms, where temperature is controlled, as is also done by Henriksson and co-workers [27]. In order to prevent strongly disturbing the system a special procedure is used to displace the tip boundary atoms. By simply moving the potential wells, the tip boundary atoms would feel strong forces and acquire high velocities. As described above, this amount of kinetic energy would be problematic for the thermostat to dissipate. Instead, we change the position of the tip boundary atoms and their potential wells simultaneously by directly adding smooth displacements. This way, they change position without additional energy transferred to the system and therefore they will not acquire high temperatures. The thermostat then only has to take care of the velocities induced by interactions with the normal atoms in the tip.

### Speed-up techniques

Several optimizations and approximations are implemented to speed up the computation so that the simulation can run in real time. First, we introduce a cutoff radius of 7 Å in the calculation of forces and energy between pairs of atoms. The exponential functions from Equation 1 are computationally heavy; a cutoff radius reduces the number of exponential functions that have to be calculated. If *r_ij_*, the distance between two evaluated atoms, is larger than the cutoff radius, the respective pair of atoms will not be taken into account in the energy and force calculations. Because of the exponential decay with distance in the potential, their contribution is very small. Moreover, as described in the book by Andrew Leach [28], just using a force cutoff would not give a decent speed-up as to use a force cutoff radius one has to compute first all the atomic distances (involving evaluating a square root, which is also computationally expensive) and then calculate the forces only within the cutoff radius. Since, in the system we study through molecular dynamics, most of the atoms do not change their nearest neighbours very often, we can avoid calculations of all distances at each time step. Instead, we introduce another cutoff radius, now for the calculation of the distances between atoms.

Moreover, we do not need to know the distance between atoms that are far apart, since their contributions will not be taken into account because of the cutoff radius for forces and energy. Therefore we only update interatomic distances at every simulation step if the previous distance was smaller than a cutoff radius of 7 Å (this second cutoff radius has to be equal to or larger than the force-cutoff radius defined earlier). The larger distances are updated less frequently, only once every 50 simulation steps. Secondly, we implement a lookup table to increase the calculation speed of the exponential functions that still need to be found. This means that the exponential function is evaluated for a long list of relevant interatomic distances at the initialization of the simulation. Every time it needs to be calculated during runtime, a linear interpolation of the precalculated values around the given distance is used instead of calculating the exponential itself. Looking up the value from the lookup table is faster than calculating it, resulting in better performance. We have compared this speed up in simulation due to the aforementioned approximations with a standard implementation of the MD simulations without any approximations. For this we performed a structural relaxation step in a system analogous to the one shown in Figure 3 and checked the difference in the final total energy of the relaxed state between our method and a conventional approach. We found that the error in final total energy induced by the cutoff radii and the lookup table is very small, approximately 10^−4^ percent (see Figure S3 in Supporting Information File 1). Using these optimization methods, a speed-up of almost 10-fold in energy and force calculation is recorded by a standard profiling tool.

A supporting program has also been developed to setup a simulation stage based on the STM images taken during the experiment prior to the start of the main program. To prepare an exact stage as in the experiments, it requires not only the exact knowledge of the positions of adatoms on the surface but also the atomic configuration of the surface and the STM tip. The atomic shape of the tip is always an unknown quantity in STM. One could obtain partial information before the start of the experiment using field ion microscopy (FIM), but after a few manipulations steps the tip shape would become unknown. We developed a localized tip-shaping procedure published elsewhere [18], which helps in preparing a crystalline tip apex up to the second atomic layer from the apex atom. In this method mechanical annealing cycles are used to achieve a more regular atomic packing. Furthermore, by imaging an adatom placed above a smooth Au surface the structure of the tip apex is imaged, and a smooth and reproducible evolution to a symmetric structure of the second layer from the tip apex atom is reported [18].

## Results and Discussion

In this section we will show how the above system with the real-time MD simulation works using some simple lateral manipulation test followed by an experiment where we lifted a chain of Au atoms out of the surface in a controlled manner forming a free-standing atomic chain between the tip and the sample. Some challenges in creating such a free-standing atomic chain using a controlled STM technique are addressed by Tartaglini and co-workers [29]. These atomic chains are ideal one-dimensional (1D) systems and are known to be formed only in pure metals such as Au, Pt and Ir. They have been studied by collecting large amounts of statistics using mechanically controlled break junction (MCBJ) [8,30] and scanning tunnelling microscope break junction (STMBJ) [31] techniques where two macroscopic size electrodes are pulled apart until the last atom contact is formed and then on further pulling of the junction new atoms from the leads join thereby forming atomic chains. From an atomistic point of view, the reason why new atoms from the bulk (where they are having more than one bond with other atoms) are pulled out to form an atomic chain can be understood by the fact that in metals the bond strength increases as the coordination number is decreased. This causes a single linear bond to become comparable to three bonds (for gold) in the bulk. Since our MD simulation uses an embedded atom potential that measures pair interactions, the effect of coordination number is automatically accounted for within the approximate atomic interaction force.

Another interesting phenomenon from the electronic point of view that was also found experimentally [8] was that the conductance of these atomic chains oscillates as a function of the number of atoms in the chain and this effect is known as ‘parity oscillations’. These oscillations were explained [32–33] as an interference effect occurring due to back-scattering of electronic waves at the interface between the bulk and the atomic chain. This back-scattering makes this phenomenon similar to that of the Fabry–Pérot interferometer in optics. This was demonstrated in experiments by making length histograms [8] of conductance and it was observed as oscillations in conductance. However, in this method averaging over many atomic-chain configurations takes place and thus such parity oscillations are smeared out. In our setup, by performing controlled lifting of a mono-atomic chain we may observe the phenomenon more clearly. Therefore free-standing atomic chains can in fact be used to test our setup, and the formation of chains can be confirmed by the observation of parity oscillations.

### Obtaining the positions of the background substrate atoms

Standard STM images of the Au(111) surface can provide the information about the crystallographic directions from the herringbone reconstruction (herringbone reconstructions have a 120° spatial symmetry and run perpendicular to the crystallographic direction 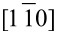 or atomically sharp step edges and the location of FCC or HCP packing. The atomic orientation and packing of metallic Au(111) surfaces is not readily available from STM images (see Figure 2b) due to the delocalized nature of the valence electrons in metals [34]. It is possible to image the atomic configuration by functionalizing the STM tip by adsorbates (for example CO molecules [35] or other foreign adsorbates [36]) at the tip apex, and by quantum point contact microscopy [37]. To keep the surface clean we did not introduce adsorbates in the system and dragging the STM tip in contact with the surface is also not a useful option as the STM tip may pick up the adatoms we want to study later.

**Figure 4:**
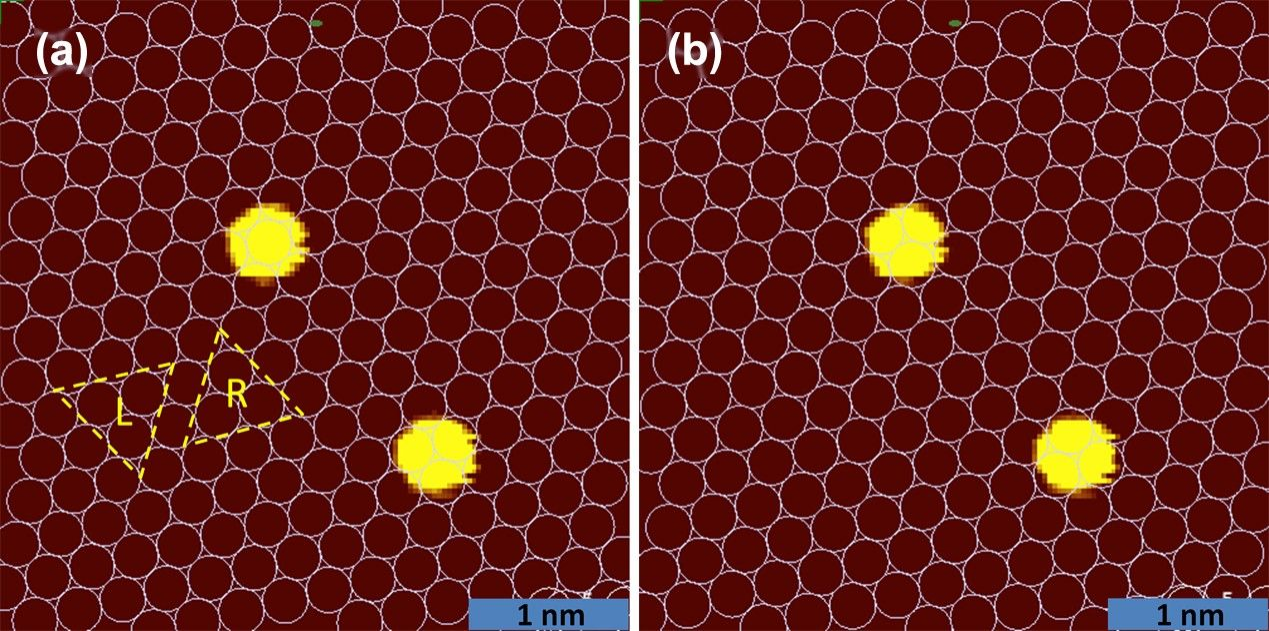
Determination of surface atom positions via geometric argumentation: The two panels show STM images with the topography colour scale tuned to reduce the apparent size of the two adatoms to match the superimposed surface lattice pattern. The full apparent size of Au adatoms on Au(111) is around 1 nm as shown in Figure 2b. (a) shows the two possible triangular hollow adsorption sites that we call ‘L’ and ‘R’ sites, depending on their orientations. It also shows that in this setting of the surface atoms one of the two adatoms sits on top of a surface, which is energetically not favourable. (b) The right image with one atom on ‘L’ and the other on ‘R’ is showing the correct positioning (energetically favourable) of the adatoms with reference to the surface lattice.

So, we devised a simple geometric technique to get the information of the surface atom positions without the need to resolve the individual surface atoms. Figure 4 shows that after fixing the crystallographic orientation (as explained above), with the use of two adatoms one can obtain with 100% certainty the complete information about the surface atoms, as the adatoms will sit only on the hollow sites (low-energy position, see Figure 4b). A detailed discussion about this is given in Supporting Information File 1.

This elegant and accurate approach allows us to determine the background lattice without the need to work towards atomic resolution of the Au(111) surface each time. The method is not limited to the Au(111) surface. A similar geometrical argument can be used on other surfaces as well, and it can be used to determine, e.g., on-top, bridge, and hollow adsorption sites of small molecules. A related approach by tracking an adatom movement and position to get the information about the background lattice has been reported earlier by Böhringer and co-workers [38]. After having determined the structure of the background lattice and the position of the adatoms with respect to it, a simulation model is constructed that has the same structure as in the experiment.

### Point contact pushing

**Figure 5:**
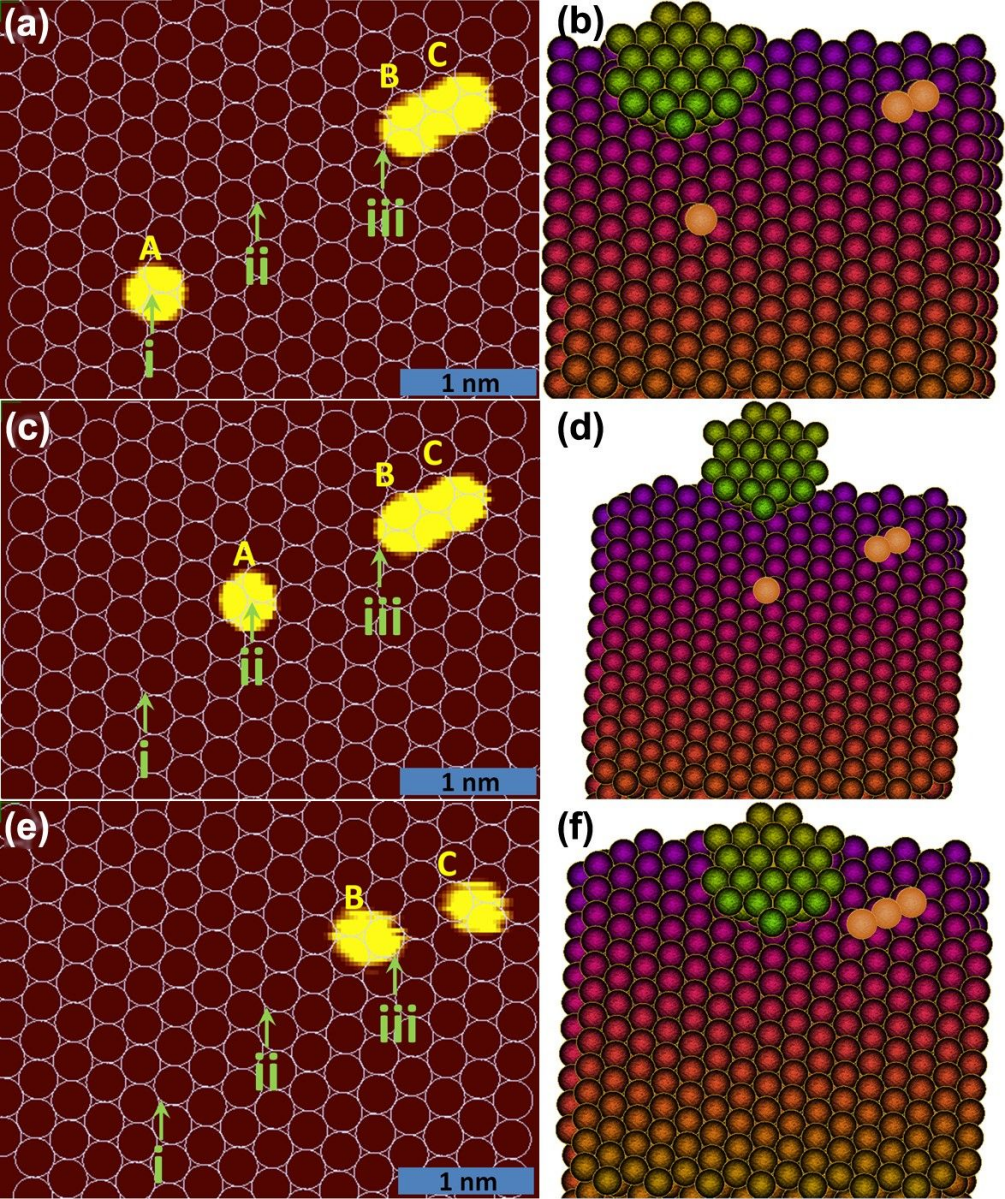
(a, c, e) show the STM images with the topography colour scale tuned to show a smaller apparent size of the adatoms ‘A’, ‘B’ or ‘C’. The scale bar in the bottom-right corner is 1 nm. The other three panels (b, d, f) show the corresponding simulation setup. (a, b) show the initial positions of adatoms ‘A’, ‘B’ and ‘C’ with ‘A’ at position ‘i’. (c ,d) show the position of adatom ‘A’ after the first move and one can see that the adatom arrives at exactly the same position ‘ii’ in both experiment and simulation. (e, f) shows the STM image and simulation after the gold atomic chain has been lifted.

We start now our experiment in the configuration shown in Figure 5a and the corresponding simulation picture in Figure 5b. An angled top view of the simulation stage is shown in the right column, where the green colour atoms are the atoms constituting the tip, while the surface is decorated in a rainbow colour scheme. This helps in better visualising the depth of the virtual scene on a 2D screen while doing the manipulation. The adatoms for the purpose of demonstration are given a false orange colour. A full sequence of the manipulation is available as a video file in the supporting information. In the left column of Figure 5 STM images obtained during different stages of the experiment are shown. Here, similar to before, the topography colour scale is tuned to show a smaller apparent size of the adatoms ‘A’, ‘B’ or ‘C’. Next, using the geometric technique explained earlier, the background atoms are determined and three fixed positions on the surface ‘i’, ‘ii’ and ‘iii’ are marked with respect to the three adatoms shown and also some other neighbouring adatoms, which are outside the field of view in the figure.

We have performed manipulation in a new point contact pushing (PCP) mode with feedback loop switched off. The difference between our PCP mode and the common lateral manipulation mode is that we do not move the tip in a straight path but we move from hollow site to hollow site by bringing the tip always in-line with the path to the next hollow site and then push the adatom. This is done so that the adatom position can be known and controlled at each step of the manipulation and avoids the complex jumps and movement of the adatom depending on the relative alignment of the underlying lattice and the manipulation direction [39]. Another important purpose this serves is that we always cross the smallest energy barrier while moving to the nearest minimum during this manipulation. Thus the inelastic energy released on snapping in this process is small, which helps also in maintaining better coordination between the simulation and the experiment.

In our manipulation method we first match the tip height in the simulation with respect to the experiment by going above an adatom in point contact in the experiment resulting in a jump to contact and a stable level at 1*G*_0_ conductance. This is different from some previous works that have shown that a jump to contact occurs only when approaching a bare metallic surface, while when approaching an adatom on the surface there is a smooth transition from tunnelling to contact [15,40]. This absence of a jump to contact has been attributed to an increased bond strength of the adsorbed atom on the surface because of the surface dipole creation due to the Smoluchowski effect. However, the authors have later reclaimed [41] that they do observe jump to contact on Au adatoms on a Au(111) surface, which they attribute to the jumping of the tip apex atom to the adatom. We have very rarely seen a smooth transition to contact and we attribute those rare events to either a blunt tip or to the presence of unwanted adsorbates that may be present in the UHV chamber, most likely hydrogen. In fact we have observed this jump to contact when approaching a Au adatom from the top in more than 80% of the times and we attribute it to the relaxation [42] of tip and surface atoms. Recent work [43–44], albeit not carried out in an STM configuration, suggests that strong relativistic effects in gold could lead to an earlier jump to contact. At this point, after matching the contact position of the tip in both simulation and experiment we retract the STM out of contact and position the tip about 1 nm “behind” the adatom, at a height corresponding to 0.4 μA tunneling current at 100 mV bias, which corresponds to a tunnel gap of 1.2 Å and around 250 kΩ tunnel resistance. Then, the tip is moved towards the adatom under feedback-off conditions while keeping *Z* constant. Similar to the jump to contact phenomenon that happens while approaching a surface or an adatom from the top, a jump to contact also occurs while approaching the adatom laterally parallel to the surface. Because the corrugation energy in metallic surfaces is usually 1/10 to 1/3 of the adsorption energy [45], this jump can even be larger in the lateral direction compared to an approach from the top.

Being thus prepared we used the PCP method to first move the adatom ‘A’ shown in Figure 5 from position ‘i’ to ‘ii’ and then a STM image (Figure 5c) is taken so that one can compare the position of the adatom in the experiment with the simulation (Figure 5d). The motion control and visual feedback from the simulation are essential in this procedure, because we need to move the tip in a zig-zag fashion behind the atom in the line of the next hollow-to-hollow hop for each lattice step. The resulting positions in Figure 5c and Figure 5d match precisely. The corresponding *x*,*y*,*z*-curves and *x*,*y*,*G*-curves for this operation are given in Supporting Information File 1 (Figure S1) and a movie showing a full sequence of this manipulation is provided as Supporting Information File 2.

Successful demonstration of manipulation using our PCP technique sets a boundary of validity of our real-time simulation. However, demonstration of the controlled lift-off of a chain of gold atoms above the Au(111) surface would depend on many different parameters that could affect the interatomic forces and are not included in simple semi-empirical potentials used here. Such parameters comprise the atomic shape of the actual tip apex in the experiment, which defines the depth of the potential well on the tip in comparison to that on the surface, and moreover the inelastic excitation of vibration modes of the adatom [46–47] due to tunnelling electrons, which could promote pick up of the adatoms from the surface. In fact, the effective atom–atom interaction employed for the simulation is a crude approximation of the true interatomic potentials, and this approximation may break down for extremely under-coordinated atoms as in atomic chains. It is also important to point out the difference between lifting monoatomic chains using STM and lifting single molecules. The atoms in the latter are mostly covalently bonded, which makes it extremely difficult to break the molecule while lifting (at least at small biases). For the atomic chains this is not the case. The interatomic bonds are much weaker and could break even at low biases. In this more demanding test of the real-time MD simulation, we will see that we obtain only partial success.

### Lifting of a gold atomic chain

**Figure 6:**
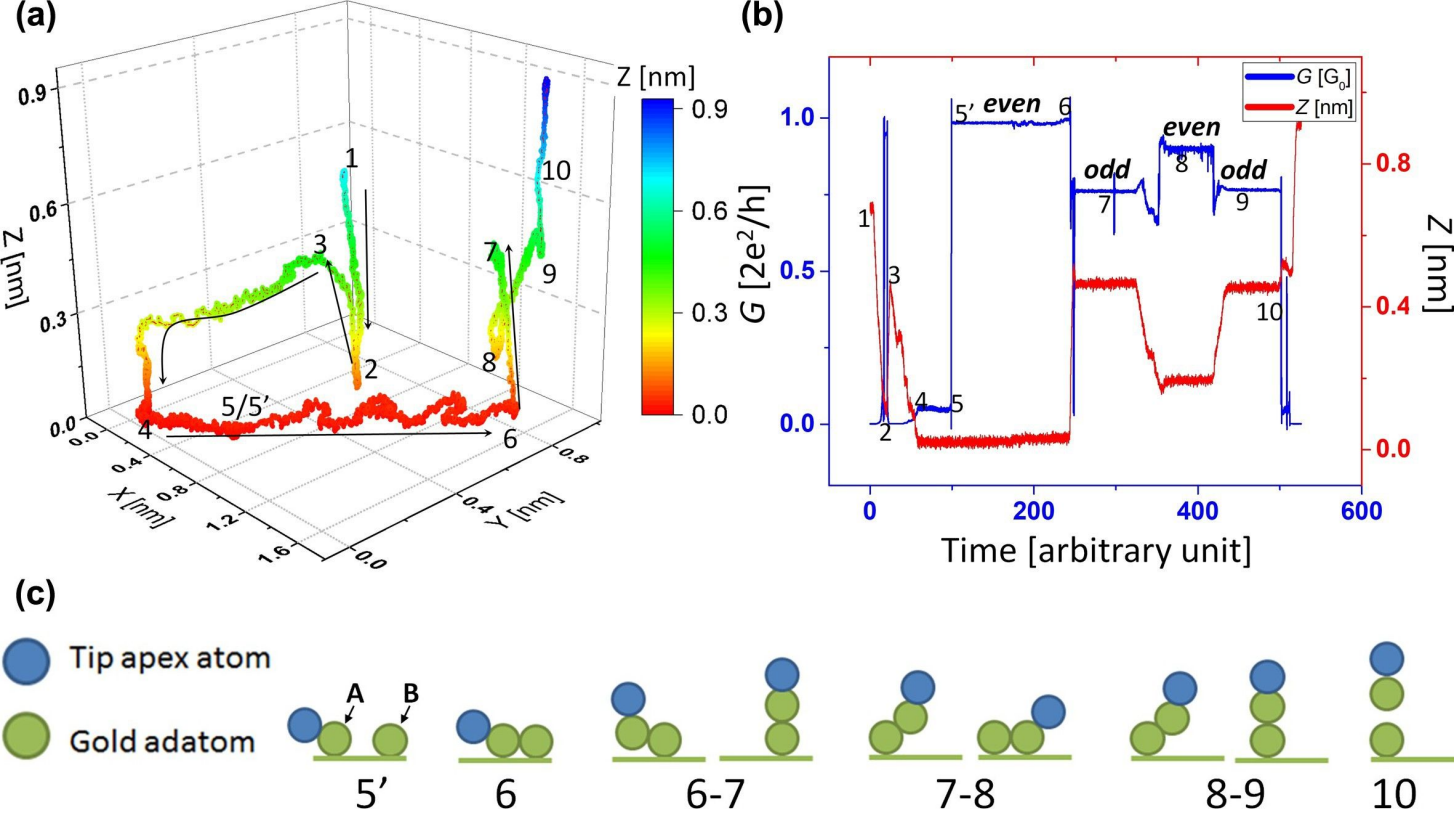
(a) Complete tip trajectory for the second step starting from position ‘ii’ in the lattice and (b) variation in conductance (blue) and *Z* coordinate (red) of the tip over the time of operation. The numbered points shown in the two graphs (a) and (b) correspond to the same points in time. (c) Schematic of the manipulation process explaining different points in the curves above. The two atoms (in green colour) here represent the ‘A’ and ‘B’ adatoms in Figure 5.

In the second step, we start now from position ‘ii’ and move the adatom ‘A’ to position ‘iii’ and then continue with the operation of lifting-off the atomic chain followed by taking an STM image at the end (Figure 5e). Figure 6a shows the tip trajectory for the second step and Figure 6b shows the corresponding conductance and *Z*-coordinate variation over time. The corresponding *x*,*y*,*G*-graph for this operation is given in Supporting Information File 1 (Figure S2) and a movie showing a full sequence of this manipulation is provided as Supporting Information File 3. The points from 1 to 10 shown in the two graphs mark the same points in time. A schematic explaining the manipulation process is given in Figure 6c. Points 1 to 4 show the beginning operation, which includes the tip height matching and positioning of the tip behind the adatom as explained in section “Point contact pushing”. After this, the tip is moved forward (keeping *Z* constant) in feedback-off state towards the adatom. Next, a lateral jump to contact happens, which gives a sudden change in conductance to approximately 1*G*_0_, as can be seen from point 5 to 5’ in Figure 6b. Note that a lateral jump to contact will also occur when adatom ‘A’ is brought closer to adatom ‘B’, but as ‘B’ and ‘C’ are very close the jump of ‘B’ towards ‘A’ should be of very short range.

Then the adatom is moved towards the other pair of atoms ‘B’ and ‘C’ to position ‘iii’ going from one hollow site to the next hollow site (‘L’ to ‘R’ or ‘R’ to ‘L’) shown as the meandering part in Figure 6a from point 5’ to 6. Now after the adatom ‘A’ has reached position ‘iii’ the tip is controllably moved from point 6 to point 7 shown in Figure 6a–b. This places the adatom ‘A’ above adatom ‘B’, which together with the tip-apex atom forms a three-atom chain as shown in Figure 6c. This causes a decrease in conductance, which can be seen clearly in Figure 6b. Note that the *Z*-position in Figure 6b shows at point 7 the *Z*-value of 0.45 nm, and adding to this 0.12 nm (which is the *Z* = 0 point given by the tip height from surface during pushing) gives 0.57 nm, which is very close to twice the covalent diameter [48] of a single Au atom (0.288 nm). After keeping the tip at this position for some time we bring the adatom ‘A’ back to the surface (point 8). The number of atoms in the chain changes back from three to two giving a step increase in conductance, shown in Figure 6b. Note that this conductance value (point 8) is lower than the earlier value between points 5’ and 6. The difference results from the fact that between point 5’ to 6 the tip is not above the adatom ‘A’, but is actually on its back in a pushing mode. Thus the overlap of the wave functions on the atomic chain and those in the tip are enhanced at this position, giving rise to a higher transmission and conductance. Then we bring the adatom ‘A’ above the adatom ’B’ (making a three-atom chain including tip apex atom) and we see again a conductance drop to exact same value as earlier (point 9 in Figure 6b).

### Parity oscillations

**Figure 7:**
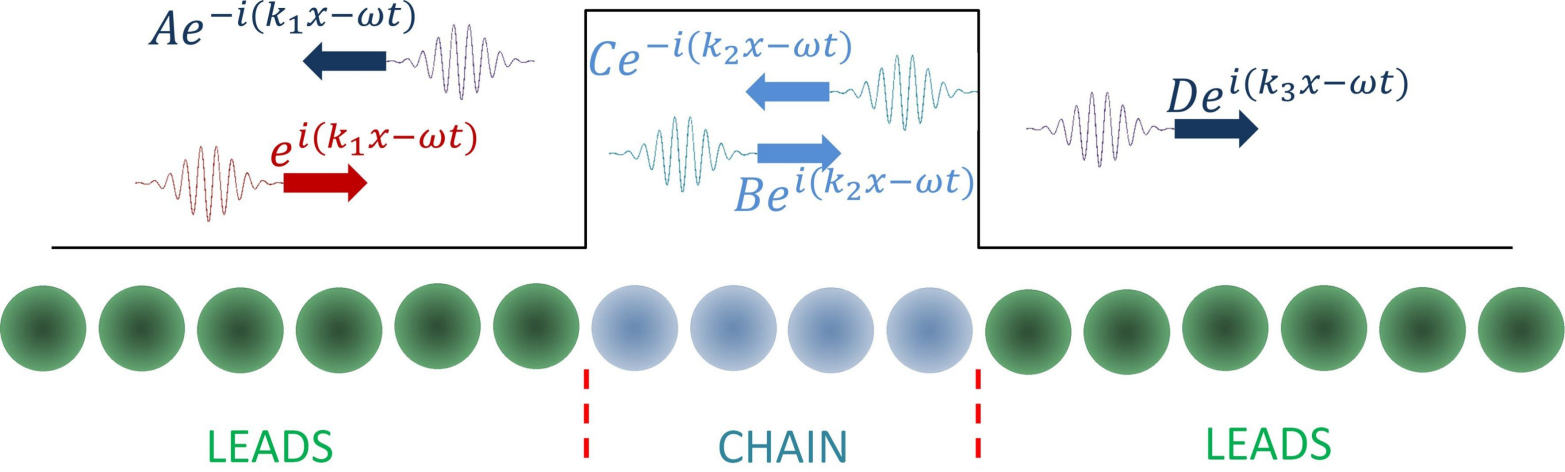
A one-dimensional model of electronic transport through a monoatomic chain [50]. To differentiate between the atomic chain and the leads, a different wave vector *k*_2_ is assigned to the chain as compared to *k*_1_ and *k*_3_ for the leads. This difference in wave vector can be manifested as a potential barrier and the electrons form standing waves inside the barrier. Depending on the length of the barrier one can have different interference patterns of these standing waves giving oscillations in the conductance. This is similar to a Fabry–Pérot interferometer. The different colours of atoms here do not imply different types of atoms but are used only to differentiate between the leads and the atomic chain.

The conductance of a macroscopic conductor decreases with increase in its length. But in small atomic-scale conductors, due to the ballistic nature of electronic transport, the conductance should not change with the length of the conductor. However, chain-length dependent oscillations in the conductance in monoatomic chains have been reported by the name of parity oscillations or even–odd oscillations. In experiments with gold atomic chains, such parity oscillations are demonstrated by making length histograms using a MCBJ setup [8,49–50]. These oscillations can be explained using a simple 1D chain model (description given in Figure 7) and its existence has been confirmed also by various detailed theoretical calculations [8,51–55]. However, there is a disagreement about the phase of these oscillations among different models. The phase defines whether the conductance of the chain with an even number of atoms will be larger than that of the chain with an odd number of atoms, or the other way around. A phase change can arise with the type of monovalent atom forming the chain (alkali metals or noble metals) [55] but can also arise due to the coupling between the electrode and the atomic chain [53–54].

The controlled experiment described in Figure 6 not only shows clearly these even–odd oscillations but also fixes their phase. We can determine here with certainty that the even number of atoms in the chain leads to a higher conductance, which agrees with the detailed calculation for Au atomic chains presented in reference [55]. As compared to previous experimental results on gold atomic chains obtained using the MCBJ technique, we have well-defined electrode shapes. One of the electrodes is an atomically flat Au(111) FCC facet and the other electrode is an atomically sharp tip apex prepared using the mechanical annealing technique [18].

Eventually, when attempting to pull the tip further expecting the adatom ‘C’ to join in the chain, the chain broke. The STM image taken at the end (Figure 5e) shows two adatoms left on the surface, supposedly the adatoms ‘B’ and ‘C’. With reference to the other neighbouring adatoms on the surface (not shown here), we can determine that the positions of ‘B’ and ‘C’ have changed but the exact sequence of steps that led to those movements cannot be determined because our simulation did not reproduce the experiment after point 6, which is not surprising. In the experiment, the adatom ‘A’ was moved above the adatom ‘B’, while in the simulation the trimer (‘A–B–C’) was left on the surface once the tip was pulled up (Figure 5f). Possible reasons why the simulation behaved differently could be, as explained earlier, the unknown shape of the tip potential well in the experiment and the excitation of substrate adatom vibration modes (at 100 mV bias) due to inelastic electron tunnelling that could promote lift-off of the chain in the experiment. In general, we have verified the agreement between simulation and experiments by comparing the result of manipulation operations. However, the mismatch also shows the inadequacy of usual semi-empirical potentials for such highly under-coordinated systems. As the actual bulk shape of the tip is unknown in our experiments we assume an isotropic tip structure, which could also cause certain discrepancies between the experiment and the simulation.

## Conclusion

We have modified our low-temperature ultra-high vacuum STM with the integration of a 3D motion control system and a real-time molecular dynamic simulation. This human–machine augmented system where the operator can become part of the experiment and make adaptable STM tip trajectories based on the visual feedback from simulation, provides a better control for (3D) atomic manipulation. This method should become particularly useful for molecular manipulation. Furthermore, we demonstrated how a simple geometric technique based on placing two adatoms can give information about the atomic configuration of the metallic substrate. We used this as the input to setup the same environment in the coupled real-time simulation and demonstrated the controlled lift-off of an atomic chain. For this purpose, we developed a new point contact pushing technique that can directly be followed by the manipulation into the third dimension at the end. By placing this atomic chain back and forth between freely suspended and surface resting position we studied the parity oscillation behaviour in conductance, which occurs due to interference of electronic waves in different length of the chain. For a better comparison with theory and a possible direct feedback loop from the experimental conductance values, a real-time conductance estimation based on the atomistic positions given by the MD simulations could be useful. Tight-binding models have been known [56] to give a relatively fast (as compared to DFT and other computationally expensive methods) estimation for the conductance values numerically. At the moment we have not added any such electronic transport model in our system but it can be a possible upgrade that should help in further guiding the experiments. It would be also interesting to study single molecules using this system. However, one has to think about the correct description of the interaction between the metallic system and the molecules. Typically MD is used for molecules in solution or for solid-state bulk materials [57], rarely for metal surfaces in contact with molecules [58]. The right way to go would be fitting a force field to experimental observations at UHV and low temperatures as in [59–60].

## Supporting Information

Supporting Information includes derivation of the force expression given in the main text. A schematic flowchart of the simulation execution is also provided. A detailed discussion about the geometric method for determining the positions of the background substrate atoms is given as well. The plot showing the effect of using the cut-off radii and look-up tables in the simulation is also provided. Conductance and trajectory data recorded during experiments are shown as 3D plots. A full sequence of the manipulation shown in the simulation is also available as a video file.

File 1 Additional data.

File 2 Simulation step 1.

File 3 Simulation step 2.
