# Supplementary material for: Intuitive human interface to a scanning tunnelling microscope: observation of parity oscillations for a single atomic chain
Source: Beilstein J Nanotechnol. 2019 Feb 4;10:337–48. doi: 10.3762/bjnano.10.33 (PMC6369976; doi:10.3762/bjnano.10.33)
Supplement: File 1 — Additional data. [file Beilstein_J_Nanotechnol-10-337-s001.pdf]

BEILSTEIN JOURNAL OF NANOTECHNOLOGY

Supporting Information

for

Intuitive human interface to a scanning tunnelling microscope: observation of parity oscillations for a single atomic chain

Sumit Tewari, Jacob Bakermans, Christian Wagner, Federica Galli
and Jan M. van Ruitenbeek

Beilstein J. Nanotechnol. **2019,** *10,* 337–348. doi:10.3762/bjnano.10.33

Additional data

License and Terms: This is a supporting information file under the terms of the Creative Commons Attribution License (http://creativecommons.org/licenses/by/4.0). Please note that the reuse, redistribution and reproduction in particular requires that the authors and source are credited.
The license is subject to the *Beilstein Journal of Nanotechnology* terms and conditions: (https://www.beilstein-journals.org/bjnano)

Force derivation

Here the force on an atom resulting from the potential energy (E) given in the main text is derived.

$$E = -\zeta \sum_{i}^{N} \sqrt{\sum_{j \neq i}^{N} e^{-2q(r_{ij}/r_0 - 1)}} + A \sum_{i}^{N} \sum_{j \neq i}^{N} e^{-p(r_{ij}/r_0 - 1)} \tag{S1}$$

Now calculating the gradient of this energy will provide the forces acting on atom a,

$$\vec{F}_a = -\vec{\nabla}_a E \tag{S2}$$

We will have to find

$$\vec{F}_a = -\vec{\nabla}_a \left[-\zeta \sum_{i}^{N} \sqrt{\sum_{j \neq i}^{N} e^{-2q(r_{ij}/r_0 - 1)}} + A \sum_{i}^{N} \sum_{j \neq i}^{N} e^{-p(r_{ij}/r_0 - 1)} \right] \tag{S3}$$

For the component k of the force ($k = x, y, z$), this becomes

$$\begin{aligned} F_{a,k} &= -\frac{\partial E}{\partial k_a} \\ &= -\frac{\partial}{\partial k_a} \left[-\zeta \sum_{i}^{N} \sqrt{\sum_{j \neq i}^{N} e^{-2q(r_{ij}/r_0 - 1)}} \right] - \frac{\partial}{\partial k_a} \left[A \sum_{i}^{N} \sum_{j \neq i}^{N} e^{-p(r_{ij}/r_0 - 1)} \right] \end{aligned}$$

$$
\begin{aligned}
&= \zeta \frac{\partial}{\partial k_a} \left[\sum_{i \neq a}^{N} \sqrt{\sum_{j \neq i}^{N} e^{-2q(r_{ij}/r_0 - 1)}} + \sqrt{\sum_{j \neq a}^{N} e^{-2q(r_{aj}/r_0 - 1)}} \right] \\
&- A \frac{\partial}{\partial k_a} \left[\sum_{i \neq a}^{N} \sum_{j \neq i}^{N} e^{-p(r_{ij}/r_0 - 1)} + \sum_{j \neq a}^{N} e^{-p(r_{aj}/r_0 - 1)} \right] \\
&= \zeta \sum_{i \neq a}^{N} \frac{\partial}{\partial r_{ia}} \sqrt{\sum_{j \neq i}^{N} e^{-2q(r_{ij}/r_0 - 1)}} \frac{\partial r_{ia}}{\partial k_a} + \zeta \frac{\partial}{\partial k_a} \sqrt{\sum_{j \neq a}^{N} e^{-2q(r_{aj}/r_0 - 1)}} \\
&- A \sum_{i \neq a}^{N} \frac{\partial}{\partial r_{ia}} \sum_{j \neq i}^{N} e^{-p(r_{ij}/r_0 - 1)} \frac{\partial r_{ia}}{\partial k_a} - A \frac{\partial}{\partial k_a} \sum_{j \neq a}^{N} e^{-p(r_{aj}/r_0 - 1)} \\
&= \zeta \sum_{i \neq a}^{N} \left[\frac{1}{2\sqrt{\sum_{j \neq i}^{N} e^{-2q(r_{ij}/r_0 - 1)}}} \left(\frac{-2q}{r_0} \right) e^{-2q(r_{ia}/r_0 - 1)} \frac{\partial r_{ia}}{\partial k_a} \right] \\
&+ \frac{\zeta}{2\sqrt{\sum_{j \neq a}^{N} e^{-2q(r_{aj}/r_0 - 1)}}} \left(\frac{-2q}{r_0} \right) \sum_{j \neq a}^{N} \left[e^{-2q(r_{aj}/r_0 - 1)} \frac{\partial r_{aj}}{\partial k_a} \right] \\
&- A \sum_{i \neq a}^{N} \left[\left(-\frac{p}{r_0} \right) e^{-p(r_{ia}/r_0 - 1)} \frac{\partial r_{ia}}{\partial k_a} \right] - A \sum_{j \neq a}^{N} \left[\left(-\frac{p}{r_0} \right) e^{-p(r_{aj}/r_0 - 1)} \frac{\partial r_{aj}}{\partial k_a} \right] \qquad \text{(S4)}
\end{aligned}
$$

where, when we take k to be x,

$$
\begin{aligned}
\frac{\partial r_{ij}}{\partial x_a} &= \frac{\partial}{\partial x_a} \sqrt{(x_i - x_j)^2 + (y_i - y_j)^2 + (z_i - z_j)^2} \\
&= \frac{2(x_a - x_j)}{2\sqrt{(x_a - x_j)^2 + (y_a - y_j)^2 + (z_a - z_j)^2}} \delta_{ia} - \frac{2(x_i - x_a)}{2\sqrt{(x_i - x_a)^2 + (y_i - y_a)^2 + (z_i - z_a)^2}} \delta_{aj} \\
&= \frac{x_a - x_j}{r_{aj}} \delta_{ia} - \frac{x_i - x_a}{r_{ia}} \delta_{aj} \qquad \text{(S5)}
\end{aligned}
$$

and similarly for y and z component, with δ_{ij} the Kronecker delta function. Substituting this in the force, we get

$$\begin{aligned}
F_{a,k} = \zeta \sum_{i \neq a}^{N} & \left[\frac{1}{2\sqrt{\sum_{j\neq i}^{N} e^{-2q(r_{ij}/r_0-1)}}} \left(\frac{-2q}{r_0}\right) e^{-2q(r_{ia}/r_0-1)} \left(\frac{k_a-k_i}{r_{ia}}\right)\right] \\
& + \frac{\zeta}{2\sqrt{\sum_{j\neq a}^{N} e^{-2q(r_{aj}/r_0-1)}}} \left(\frac{-2q}{r_0}\right) \sum_{j\neq a}^{N} \left[e^{-2q(r_{aj}/r_0-1)} \left(\frac{k_a-k_j}{r_{aj}}\right)\right] \\
& - A \sum_{i\neq a}^{N} \left[\left(-\frac{p}{r_0}\right) e^{-p(r_{ia}/r_0-1)} \left(\frac{k_a-k_i}{r_{ia}}\right)\right] - A \sum_{j\neq a}^{N} \left[\left(-\frac{p}{r_0}\right) e^{-p(r_{aj}/r_0-1)} \left(\frac{k_a-k_j}{r_{aj}}\right)\right] \\
& = -\zeta \frac{q}{r_0} \sum_{i\neq a}^{N} \left[\frac{e^{-2q(r_{ia}/r_0-1)}}{\sqrt{\sum_{j\neq i}^{N} e^{-2q(r_{ij}/r_0-1)}}} \left(\frac{k_a-k_i}{r_{ia}}\right)\right] \\
& - \frac{\zeta(q/r_0)}{\sqrt{\sum_{j\neq a}^{N} e^{-2q(r_{aj}/r_0-1)}}} \sum_{j\neq a}^{N} \left[e^{-2q(r_{aj}/r_0-1)} \left(\frac{k_a-k_j}{r_{aj}}\right)\right] + 2A\frac{p}{r_0} \sum_{j\neq a}^{N} e^{-p(r_{aj}/r_0-1)} \left(\frac{k_a-k_j}{r_{aj}}\right)
\end{aligned} \tag{S6}$$

Geometric method for determining the positions of the background substrate atoms

At the start of the experiment we deposit single Au ad-atoms on the middle of the FCC sector of the herringbone reconstruction on a clean Au(111) surface with the STM tip as explained earlier (see Experimental Setup section in the main text). Then a simulation has to be prepared with the ad-atoms lying on the exact same atomic configuration on the surface as in the e xperiment. To get the information about the background atoms (without functionalizing the tip), we use a geometry based technique. After our surface preparation we can get directly from the orientations of the straight step edges (Figure 2(a) in the main text) the angle of the three crystallographic directions (shown with white lines) in our STM images. These steps appear after locally distorting the surface by tip indentation at a place remote from the measurement location. Once the three crystallographic directions are established the next step is to find out whether the ad-atom sits on the left (L) or right (R) triangular hollow site (shown in Figure 4(a) in the main text). Note the crystallographic directions of

the surface locally can also be obtained using the angles of the herringbone reconstruction in (111) surfaces provided that the location is far from any defect sites or corners of the herringbone as these can change or alter the orientation locally. A crystallographic layer formed by ad-atoms adsorbed on either of the two sites would form a FCC or HCP packing along with the present top two layers. This is the reason the two triangular hollow adsorption sites are formally called FCC or HCP sites. But without the prior knowledge of the top two layers, we cannot tell which site is FCC or HCP. So, we prefer to call them 'L' and 'R' sites, depending on their orientations.

Within a linear translation of the lattice, there is a 50% chance of being correct if we choose one of the two hollow sites (L or R). But in fact with the help of just two ad-atoms and a geometric argument we could tell with 100% certainty the position of both the ad-atoms and have a complete information about the surface atoms. If we take any two ad-atoms placed near to each other in the experiment, they could be lying either on similar orientated hollow sites (LL or RR) or oppositely oriented hollow sites (LR or RL). We overlay the measured STM image with the known lattice periodicity, as shown in Figure 4. If they are placed on oppositely oriented hollow sites (as shown in Figure 4(b)), then within the translational symmetry of the surface lattice there is only one possible orientation : either we find that the top left atom sits in the L site and the lower right one in the R site, or the other way around. For example if we try to translate the surface lattice scheme such that the bottom right ad-atom in Figure 4(b) shifts from the R hollow site (where it is lying) to a L hollow site as shown in Figure 4(a), then one can see that the top left ad-atom does not fit anymore in any hollow site. This shows that only the positioning of the ad-atoms shown in Figure 4(b) is correct. On the other hand if they are placed on similar hollow sites (LL or RR), then it is not possible to directly conclude their positions by doing the linear translation described above. The solution, then, is to perform one atomic manipulation move of one of the two ad-atoms to bring them in the oppositely oriented hollow site.

Lifting of gold atomic chain

In the article a controlled formation of free standing atomic chain between STM tip and sample is demonstrated. The total operation is divided into two steps. In first step the ad-atom ‘A’ (marked in Figure 5 in the main text) is pushed from position ‘i’ to ‘ii’ and then a STM image is taken (Figure 5(c) in the main text). The corresponding x,y,z and x,y,G curves for this operation are given here in Figure S1.

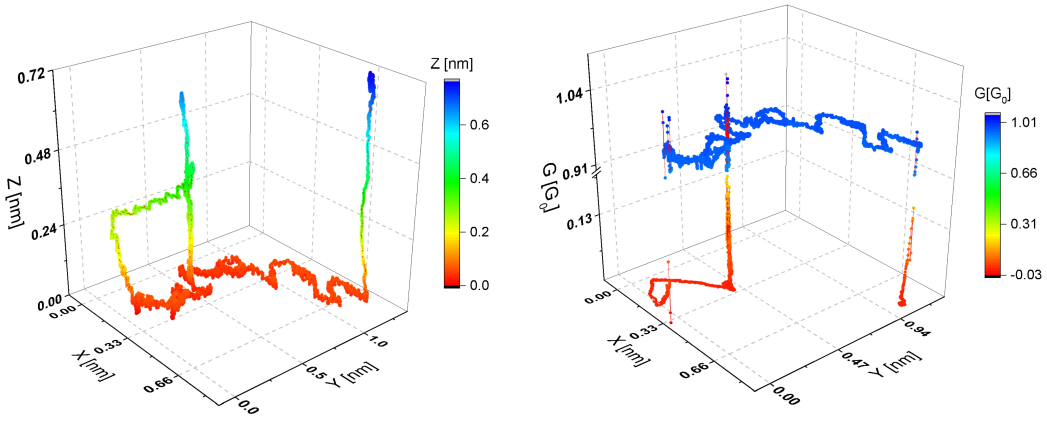

(a) (b)

Figure S1: (a) Complete tip trajectory for the first step starting from position ‘i’ in the lattice and (b) variation in conductance over the time of operation.

In the second step we start from position ‘ii’ and move the ad-atom ‘A’ to position ‘iii’ and then continue with lifting of the atomic-chain followed by taking the STM image at the end (Figure 5(e) in the main text). Figure S2(a) shows the tip trajectory for the second step and Figure S2(b) shows the corresponding conductance variation over the time.

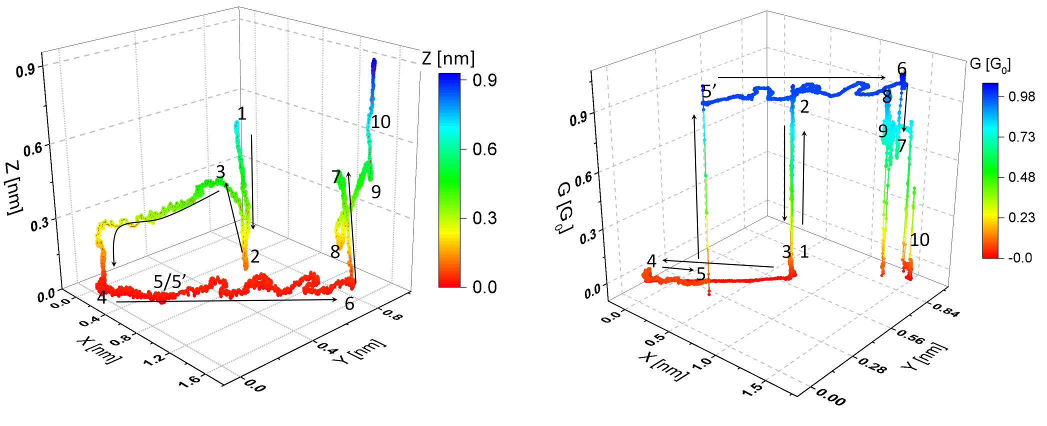

(a) (b)

Figure S2: (a) Complete tip trajectory for the second step ending in a lift-off of a mono-atomic gold chain from the Au(111) surface and (b) variation in conductance during the lift-off of the atomic chain.

Simulation movies are also provided as a supplementary information. These are movies from a single view angle, however during the actual manipulation the operator has an access to alter the view angle in real time.

Effect of using cut-off radii and look-up tables in the simulation

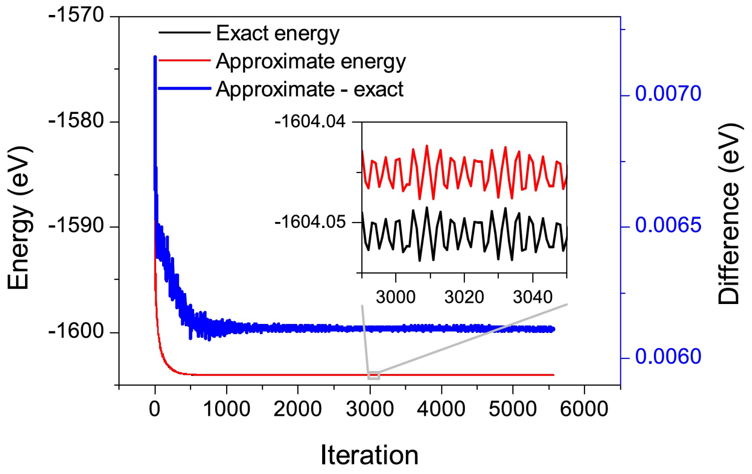

Figure S3: Comparison between the energy calculated with (red) and without (black) approximations during relaxation of the structure without tip motion. The calculated energy is plotted against the simulation iteration number during tip and surface relaxation with the energy scale on the left. The difference between the two (approximate energy minus exact energy) is plotted in blue with the energy scale on the right. A strongly zoomed-in cut-out is shown for the energy in the relaxed configuration.

Simulation Structure

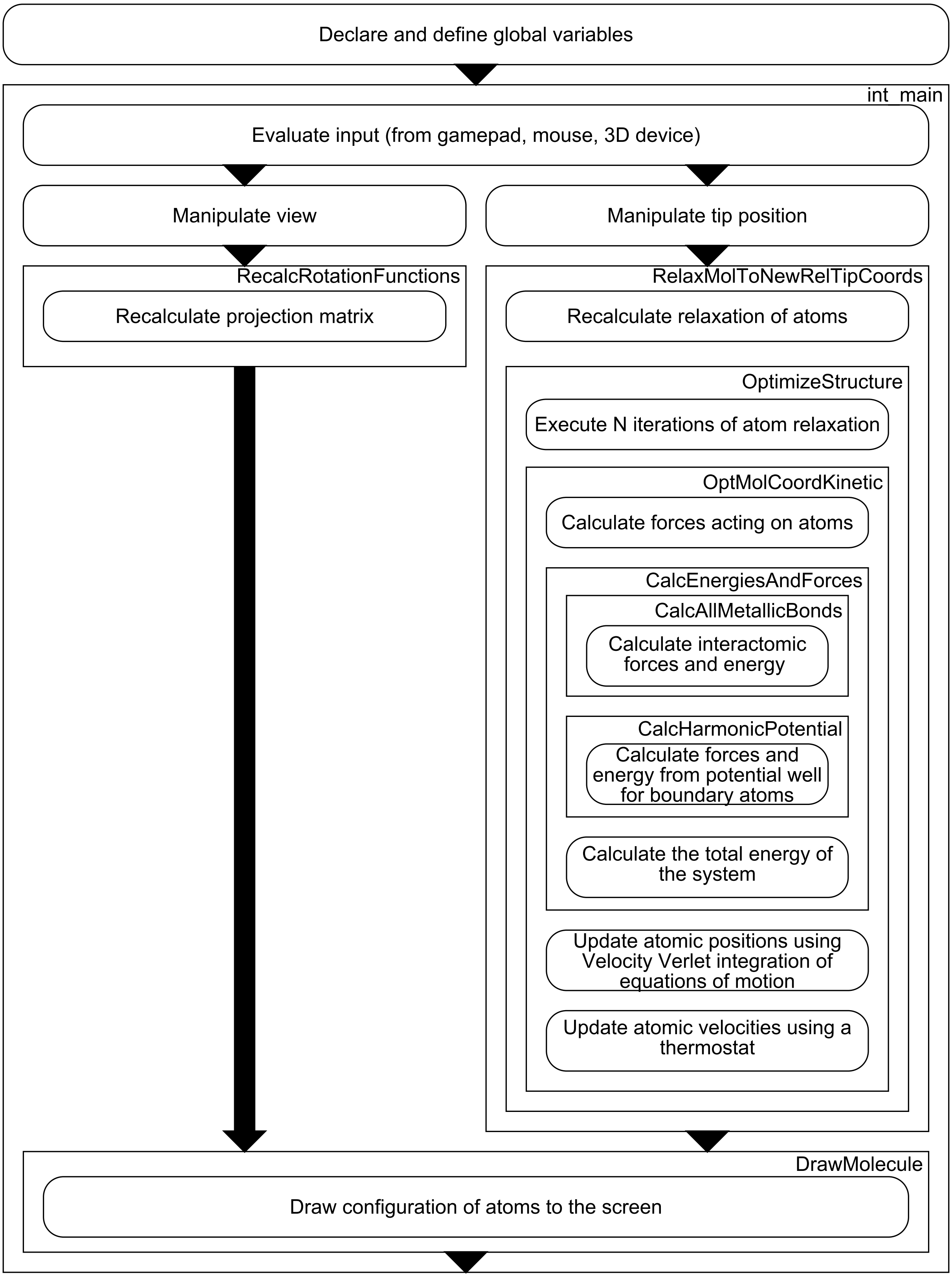

Figure S4: Schematic flowchart of simulation execution. Functions are represented by rectangular boxes with their name in the upper right corner. The actions they perform are shown in the boxes with round edges.
